# Supplementary material for: Persistent mosquito fogging can be detrimental to non-target invertebrates in an urban tropical forest
Source: PeerJ. 2020 Oct 1;8:e10033. doi: 10.7717/peerj.10033 (PMC7533057; doi:10.7717/peerj.10033)
Supplement: Supplemental Information 1 — Each GPS coordinates correspond to where the fogging experiments were carried out in Kota Damansara Community Forest (KDCF). [file peerj-08-10033-s001.docx]

| **Kota Damansara Community Forest (KDCF)** | **GPS coordinates** |
| --- | --- |
| Site 1 | N 03°10.206’, E 101°34.886’ |
| Site 2 | N 03°10.219’, E 101°35.101’ |
| Site 3 | N 03°10.158’, E 101°35.046’ |
| Site 4 | N 03°10.104’, E 101°35.063’ |
| Site 5 | N 03°10.066’, E 101°35.019’ |
| Site 6 | N 03°10.014’, E 101°35.012’ |
| Site 7 | N 03°09.969’, E 101°35.043 |
| Site 8 | N 03°10.024’, E 101°35.049’ |
| Site 9 | N 03°10.071’, E 101°35.130’ |
| Site 10 | N 03°10.053’, E 101°35.197’ |
