# Supplementary material for: Persistent mosquito fogging can be detrimental to non-target invertebrates in an urban tropical forest
Source: PeerJ. 2020 Oct 1;8:e10033. doi: 10.7717/peerj.10033 (PMC7533057; doi:10.7717/peerj.10033)
Supplement: Supplemental Information 2 [file peerj-08-10033-s002.pdf]

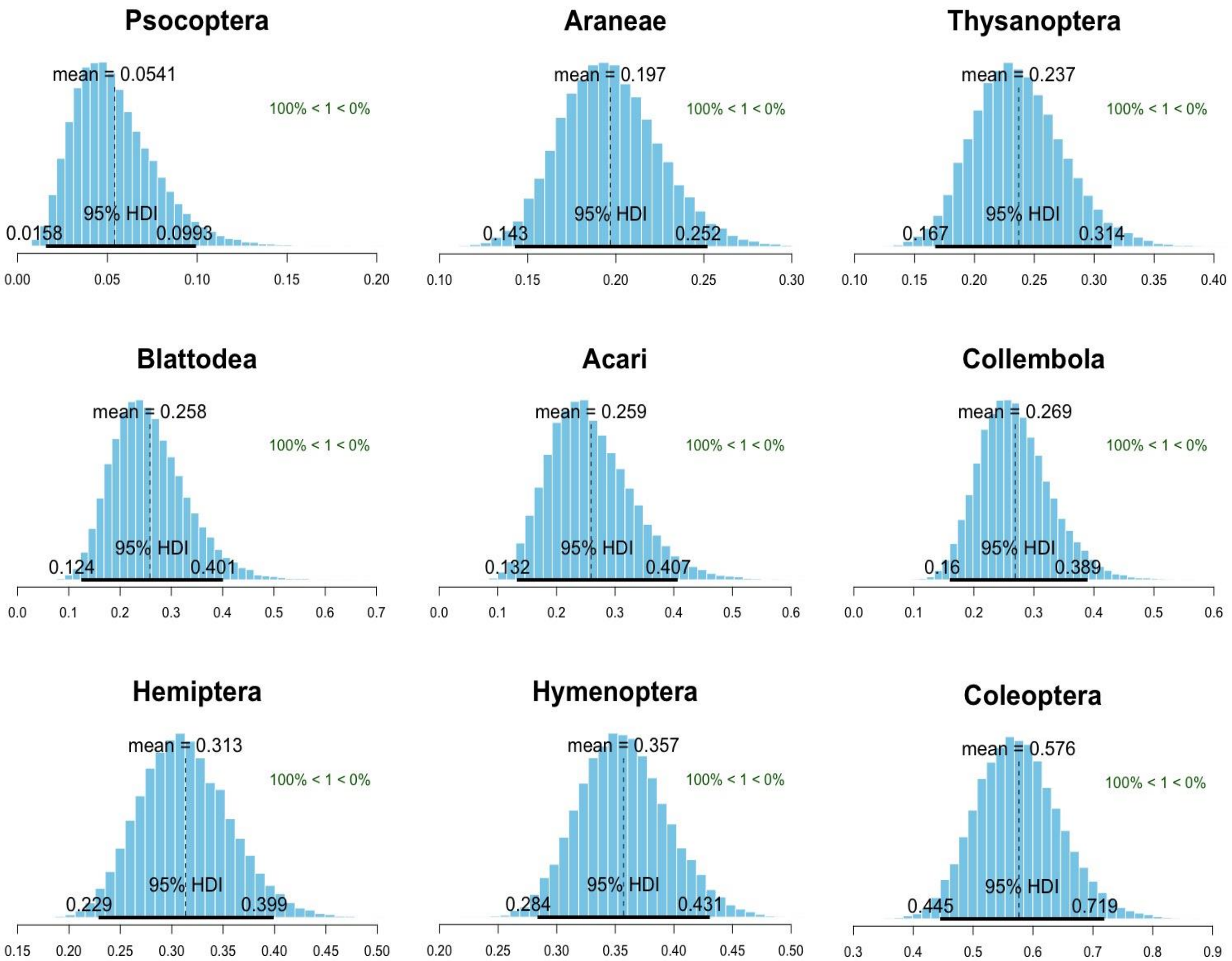

Psocoptera
mean = 0.0541
100% < 1 < 0%
95% HDI
0.0158
0.0993
0.00 0.05 0.10 0.15 0.20
Araneae
mean = 0.197
100% < 1 < 0%
95% HDI
0.143
0.252
0.10 0.15 0.20 0.25 0.30
Thysanoptera
mean = 0.237
100% < 1 < 0%
95% HDI
0.167
0.314
0.10 0.15 0.20 0.25 0.30 0.35 0.40
Blattodea
mean = 0.258
100% < 1 < 0%
95% HDI
0.124
0.401
0.0 0.1 0.2 0.3 0.4 0.5 0.6 0.7
Acari
mean = 0.259
100% < 1 < 0%
95% HDI
0.132
0.407
0.0 0.1 0.2 0.3 0.4 0.5 0.6
Collembola
mean = 0.269
100% < 1 < 0%
95% HDI
0.16
0.389
0.0 0.1 0.2 0.3 0.4 0.5 0.6
Hemiptera
mean = 0.313
100% < 1 < 0%
95% HDI
0.229
0.399
0.15 0.20 0.25 0.30 0.35 0.40 0.45 0.50
Hymenoptera
mean = 0.357
100% < 1 < 0%
95% HDI
0.284
0.431
0.20 0.25 0.30 0.35 0.40 0.45 0.50
Coleoptera
mean = 0.576
100% < 1 < 0%
95% HDI
0.445
0.719
0.3 0.4 0.5 0.6 0.7 0.8 0.9
